# Fast exchange fluxes around the pyruvate node: a leaky cell model to explain the gain and loss of unlabelled and labelled metabolites in a tracer experiment

**DOI:** 10.1186/s40170-016-0153-9

**Published:** 2016-07-04

**Authors:** Lake-Ee Quek, Menghan Liu, Sanket Joshi, Nigel Turner

**Affiliations:** Department of Pharmacology, School of Medical Sciences, UNSW Australia, Sydney, NSW 2052 Australia; The Charles Perkins Centre, School of Mathematics and Statistics, The University of Sydney, Sydney, NSW 2006 Australia

**Keywords:** 13C tracers, Metabolic flux analysis, Lactate, Pyruvate, Extracellular metabolites, Metabolite exchange, GC-MS

## Abstract

**Background:**

Glucose and glutamine are the two dominant metabolic substrates in cancer cells. In ^13^C tracer experiments, however, it is necessary to account for all significant input substrates, as some natural (unlabelled) substrate in the medium, often derived from serum, can be metabolised by cells despite not showing signs of net consumption.

**Results:**

Using [U-^13^C_6_]-glucose tracers and measuring extracellular metabolite enrichments by GC-MS, we found that pancreatic cells HPDE and PANC-1 secrete lactate, pyruvate, TCA cycle metabolites and non-essential amino acids synthesised from glucose. Focusing our investigations on pyruvate exchange in HEK293 cells, we observed that the four metabolites pools, intracellular and extracellular lactate and pyruvate, had similar ^13^C enrichment trajectories. This indicated that these metabolites can mix rapidly. Using a hybrid ^13^C-MFA, we followed to show that the lactate exchange flux had increased when extracellular lactate concentration was increased by 10-fold. By allowing rapid exchange fluxes around the pyruvate node, ^13^C-MFA revealed that PANC-1 cells cultured in [U-^13^C_6_]-glucose doubled the conversion of unlabelled substrates to pyruvate when treated with TNF-α.

**Conclusions:**

The current work established the possibility that a cell’s range of significant input substrates may be broader than anticipated. Metabolite exchange can affect intracellular enrichments. In particular, we showed that pyruvate was more strongly connected to lactate than to upstream glycolytic intermediates and that a fast lactate exchange may alter the outcome of flux analyses. Nevertheless, the leaky cell model may be an opportunity in disguise—the ability to continuously monitor metabolism using only the enrichments of extracellular metabolites.

**Electronic supplementary material:**

The online version of this article (doi:10.1186/s40170-016-0153-9) contains supplementary material, which is available to authorized users.

## Background

The metabolism of cancer cells is heterogeneous and highly adaptive [1, 2]. Indeed, many recently discovered metabolic features of cancer cells have been unexpected, notably the alternative glycolysis by phosphoglycerate mutase 1 and pyruvate kinase M2 isoform [3] and the simultaneous oxidative and reductive conversion of 2-oxoglutarate [4]. Because of the extensive scope of metabolic rewiring in cancer cells, there is intense interest in investigating the potential of specific metabolic enzyme and pathways as therapeutic targets [5–7].

Metabolomics and metabolic tracers have been an invaluable tool in understanding complex metabolic alterations that occur in response to physiological stressors and disease [8–10]. Targeted metabolite profiling, via very elegantly designed tracer experiments and/or flux modelling, have provided a read-out of how metabolic pathways are utilised/altered in cancer [4, 11–14]. Commonly, tracer experiments have been conducted on the premise that glucose and glutamine are the two dominant substrates [15]. The aim is to resolve and understand the re-routing of these nutrients caused by certain metabolic perturbations, to fulfil energy, biosynthesis and redox demands [16]. Pathway activity interpretations are made under the context of a metabolic model, often rendered simple but still physiologically representative.

Metabolic flux analysis is a very useful modelling technique to quantify metabolism [17, 18], but its translation from microbial to mammalian system has been hindered by mammalian cells’ fastidious nutritional demands, e.g., serum. In a serum- and protein-free bioreactor culture, about 65 and 10 % of the carbon uptake flux of CHO-K1 (Chinese hamster ovary) cells were attributed to glucose and glutamine, respectively, with amino acids constituting the balance [19]. The same experiment, using [U-^13^C_6_]-glucose, also showed the reversible exchange of labelled pyruvate, lactate and amino acids (alanine, serine, glycine, aspartate, glutamate and glutamine) between intracellular and extracellular pools. The rapid exchange of amino acids was also described in protein-scavenging PDAC (human pancreatic ductal adenocarcinoma) cells [20]. CORE (cellular consumption and release) profile of metabolites in the NCI-60 panel showed two thirds of 111 metabolites were consistently released into the medium, and these included glycolytic and TCA (tricarboxylic acid) cycle intermediates [21]. While not all of these exchanges have significant metabolic flux contribution relative to glucose and glutamine, the significant ones will affect tracer-based flux modelling. Therefore, it is necessary to identify and specify these fluxes upfront.

Our investigation was spurred by frequent unexpected observations that pyruvate and lactate were barely labelled by [U-^13^C_5_]-glutamine in carbon tracer experiments. Often, it is assumed that malic enzymes are an integral part of glutaminolysis, converting malate to pyruvate [13, 22, 23]. However, when applying [U-^13^C_5_]-glutamine to ovarian cancer cells [12], myoblasts [24], PDAC cells [13], and even CHO cells [25], pyruvate/lactate pools were by and large unlabelled, despite malate and TCA metabolites being predominantly labelled by glutamine. It is possible that many pathways that converge at pyruvate, in addition to glycolysis, have fluxes greater than the malic enzymes. With [U-^13^C_6_]-glucose, lactate not only showed a slower enrichment dynamic compared to phosphoenolpyruvate but the enriched amount was halved [25, 26]. In a [U-^13^C_5_]-glutamine experiment, M3 (fully labelled) lactate continued to increase linearly over 300 min despite M4 malate peaking at 100 min, although the fraction of M3 lactate was less than 1 % [13]. Both cases demonstrated that the connection of pyruvate with glycolysis and glutaminolysis is weaker than anticipated. Why glutaminolysis, a dominant contributor to TCA cycle flux, had such a small contribution to pyruvate formation, is unclear.

It is crucial to identify all significant pathways that converge at a metabolite node when using tracer data to infer metabolic activity. Particularly for cancer cell models, dominant metabolic features like glycolysis and glutaminolysis are included by default, but unrelated pathways are often ignored to simplify analysis. In the current study, enriched TCA cycle metabolites were found to be present extracellularly in PDAC and HPDE (human pancreatic duct epithelial) cells fed with [U-^13^C_6_]-glucose. This may explain the gradual loss of labelled glutamine observed in previous work and drew attention to the possibility that intracellular metabolites can exchange with extracellular pools.

Thus, we hypothesised that high exchange fluxes—a “leaky” cell—caused the dilution of ^13^C labelling at the pyruvate node. We showed that high extracellular lactate can dampen the enrichment rate of intracellular pyruvate due to rapid inter-compartment exchanges. In other words, pyruvate enrichment is not solely dependent on glycolysis and glutaminolysis, but on surrounding exchange fluxes as well. On top, we showed that a “leaky” cell model, despite making flux analysis more complicated, can be advantageous as a convenient approach to continuously monitor intracellular metabolic activity using extracellular enrichments.

## Methods

### Reagents and cells

All chemical standards (glucose, sodium lactate, sodium pyruvate) and reagents were purchased from Sigma-Aldrich (Castle Hill, Australia), unless indicated otherwise. HPLC-grade methanol and chloroform were used. [U-^13^C_6_]-glucose was purchased from Sigma-Aldrich (Castle Hill, Australia). Succinic acid-d6 (99 atom % D) from MSD Isotopes (Montreal, Canada) was kindly provided by BMSF (UNSW Australia). Recombinant human TNF-α was purchased from R&D systems (Minneapolis, MN, USA). Dialysed foetal calf serum (Life Technologies) was kindly provided by Holst Lab (USYD Australia).

HEK 293 and the PDAC cell line PANC-1 cells were cultured in Dulbecco’s modified Eagle’s medium containing 1 and 4.5 g/L glucose (Sigma-Aldrich), respectively, supplemented with 10 % foetal bovine serum (FBS, Gibco) and penicillin-streptomycin (Gibco). HPDE cells (a kind gift from Dr. Darren Saunders, UNSW Australia) were cultured in keratinocyte serum-free media (Gibco) supplemented with epidermal growth factor (5 ng/ml) bovine pituitary extract (50 ug/ml) and penicillin-streptomycin (Gibco).

### HEK cell culture in EM and FM media

HEK 293 cells were cultured in 13 × 6-cm cell culture dishes in a humidified incubator set at 37 °C and 5 % CO_2_. Cells were seeded such that each dish contained 1 million cells after 40 h of incubation. One of the dishes was used for cell count (total 1.12 million cells). At the start experiment, cell culture media from six dishes were combined. [U-^13^C_6_]-glucose was then added to reach 1 g/L in concentration, and 2 ml of the labelled existing medium (EM) was transferred back to the dishes. For the remaining six dishes, cell culture medium was replaced with 2 ml of fresh medium (FM) containing an additional 1 g/L [U-^13^C_6_]-glucose. A dish each from the EM and FM groups was harvested every half hour for cells and cell culture medium.

### PANC-1 cells treated with TNF-α

PANC-1 cells were seeded in 6-well plates at a density of 1.5 × 10^5^ per well and treated with either 40 ng/ml TNF-α or phosphate-buffered saline for 72 h. Cell culture medium was then changed for 2 ml (per well) of DMEM containing 4.5 g/L [U-^13^C_6_]-glucose. From one well of the control and TNF-α, 50 μl of cell culture medium was sampled every hour for 5 h. Cells from three other parallel wells were harvested for protein abundance at midpoint (2.5 h) and lysed with RIPA buffer pH 7.5 containing 20 mM Tris-HCl, 150 mM NaCl, 1 mM EDTA, 1 mM EGTA, 1 % NP-40 1 % sodium deoxycholate, 2.5 mM sodium pyrophosphate and 1 mM b-glycerophosphate for protein content determination. Protein concentration of the lysates was determined using the Pierce BCA assay kit (Thermofisher Scientific, USA).

### HPDE and PANC-1 cell culture in glucose-labelled medium

HPDE and PANC-1 cells were seeded in 6-well plates at a density of 3 × 10^5^ per well and cultured at 37 °C with 5 % CO_2_ for 48 h. Media from each well was then replaced with 2 ml of glucose-free DMEM, with 10 % FBS, containing 1 g/L of either normal glucose or [U-^13^C_6_]-glucose; 1.5 ml of cell culture medium was harvested after a 24-h incubation.

### Metabolite sampling and extraction

For extracellular samples, harvested cell culture medium was centrifuged at 300*g* (4 °C) for 5 min, with the supernatant stored in −30 °C freezer until analysis.

For intracellular samples, the remaining medium was removed before washing each dish once with 5 ml of ice-cold 0.9 % w/v NaCl (saline) solution. Metabolites were then extracted using 2.5 ml of 50 % *v*/*v* methanol:water mixture at −30 °C. Cells were scraped in this mixture before being transferred into a 15-ml falcon tube kept in ice. The plate was rinsed once with 2.5 ml of ice-cold Milli-Q water, and the solution was combined with the first extract; 5 ml of chloroform at −30 °C was added to the extraction mix, followed by 10 s of vortexing and 5 min of centrifuging at maximum speed. The aqueous phase was transferred into a glass tube and evaporated to dryness without heat by SpeedVac (Savant). Dried samples were promptly derivatised.

### MAB derivatization

**Fig. 1:**
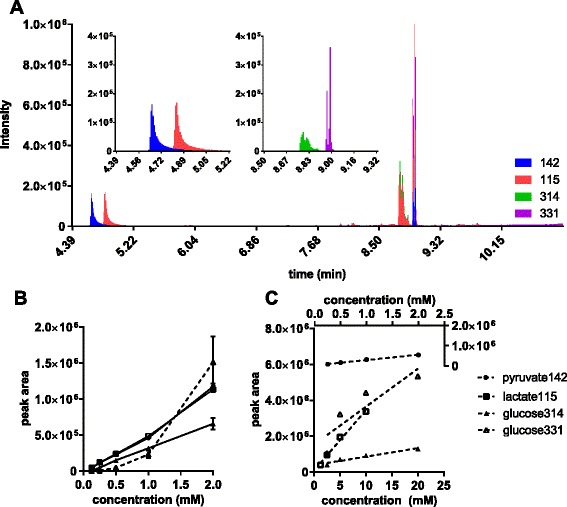
GC-MS quantification of metabolites derivatised by methoximation-acetylation-butylation. The volume of derivatised standard mixture was 10 μl, the injection volume was 1 μl splitless, and ions were monitored with a dwell time of 50 ms. Peaks areas were normalised to internal standard succinic acid-d6. **a** GC chromatogram generated in scan mode for equimolar (2 mM) standard of pyruvate, lactate and glucose. Pyurvate and lactate peaks are shown as ions 142 and 115, respectively (*left zoom-in panel*). The methoxime and nitrile derivatives of glucose pentaacetate peaks are shown as ions 331 and 314, respectively (*right zoom-in panel*). **b** Equimolar calibration curves for pyruvate, lactate and glucose. Duplicate injection of standards between 0.125 and 2 mM. **c** Similar calibration curves with concentrations set at expect medium ranges: 0.25–2.0 mM for pyruvate, 1.25–10 mM for lactate, and 2.5–20 mM for glucose

We combined three different derivatisation strategies into a one-pot reaction synthesis: methoximation, aldonitrile peracetate derivatization [27] and alkylation using chloroformate [28, 29] (see Additional file 1: S4). Methoxyamine hydrochloride, which often is used in conjunction with silylation, reacts with aldehyde and ketone functional groups to prevent keto-enol tautomerization. Subsequent addition of acetic anhydride acetylates the alcohol group of lactate and glucose. Finally, the addition of butanol and chloroformate leads to butylation of the carboxylic group of lactate and pyruvate. This method was used to derivatise all longitudinal samples extracellular lactate, pyruvate and glucose because the GC programme is significantly shorter (<11 min) (Fig. 1a).

The following describes the procedure used for methoximation-acetylation-butylation (MAB) derivatization; 10 μl of the thawed supernatant was combined with 10 μl of succinic acid-d6 (10 mM) in a glass vial and was evaporated to dryness by SpeedVac. Dried samples were resuspended in 15 μl of pyridine containing 20 mg/ml methoxyamine HCl and then incubated at 80 °C for 1 h; 15 μl of acetic anhydride was added, followed by another hour of incubation at 80 °C. Once cooled to room temperature, 50 μl of 1-butanol and 10 μl of ethyl chloroformate were added in succession, with each step followed by brief vortexing. Samples were kept at room temperature for 5 min before being transferred into 600-μl microcentrifuge tubes; 80 μl of chloroform was added, followed by 10–15 mg of sodium hydrogen carbonate solids and 75 μl of saturated sodium hydrogen carbonate solution. The organic and aqueous phases were mixed by pipetting. After the bubbling had ceased, a further 150 μl of saturated sodium hydrogen carbonate solution was added. After brief vortexing, samples were centrifuged at 500*g* for 5 min. About 70 μl of the chloroform (bottom) phase was transferred into GCMS vials using gel-loading pipet tips.

Two sets of glucose, lactate and pyruvate external standards were prepared in twofold serial dilutions (Fig. 1b, c). The first set had a starting concentration of 2 mM for all metabolites (equimolar series); the second set had a starting concentration of 20, 10, and 2 mM for glucose, lactate and pyruvate, respectively (cell culture ranges); 10 μl from each standard mixture was combined with 10 μl of succinic acid-d6 (10 mM) in a glass vial, and the solutions were dried and derivatised as describe previously. Analyte responses were linear for pyruvate and lactate (r2 > 0.985). Glucose signals, however, showed a weaker linear correlation to concentrations, with the nitrile derivative (glucose 314) showing a more consistent response (r2 > 0.95) than the methoxime derivative (glucose 331) (r2 < 0.84). The gluconitrile derivative was used for flux calculations.

### Silylation of metabolites

For tBDMS derivatization, 40 μl of pyridine containing 20 mg/ml methoxyamine HCl was added to dried intracellular metabolites, followed by 80 °C incubation for 1 h; 30 μl of MTBSTFA + 1 % t-BDMCS was then added, followed by another hour incubation at 80 °C. Derivatised samples were then transferred into GCMS vials.

For TMS derivatization, 40 μl of pyridine containing 20 mg/ml methoxyamine HCl was added to dried standards and cell culture media prepared in GCMS vials, followed by 40 °C incubation for 1.5 h; 30 μl of MSTFA was then added, followed by 0.5-h incubation at 40 °C.

### GCMS

Derivatised metabolites were analysed by GC-MS using a HP-5ms capillary column (0.25 mm i.d. × 30 m × 0.25 μm; Agilent J&W) installed in an Agilent HP 6890-5973 gas chromatography/mass selective detector. The injection volume was 1 μL in splitless mode with an inlet temperature of 250 °C. Helium flow was controlled at 1.1 ml/min. The MS was operated in electron ionisation mode at 70 eV. The temperatures of the source, quadrupole and the transfer line were set at 150, 230 and 250 °C, respectively. For tBDMS metabolites, the GC temperature programme was 70 °C for 2 min, ramp at 4 °C/min to 200 °C and at 15 °C/min to 290 °C, then hold for 6 min. For TMS metabolites, the GC temperature programme was 70 °C for 2 min and ramp at 4 °C/min to 203 °C, then bake-out for 10 min at 299 °C. For MAB metabolites, the GC temperature programme was 100 °C for 2 min, ramp at 15 °C/min to 150 °C and at 40 °C/min to 325 and hold for 1.3 min.

### Tracer modelling and computation

**Fig. 2:**
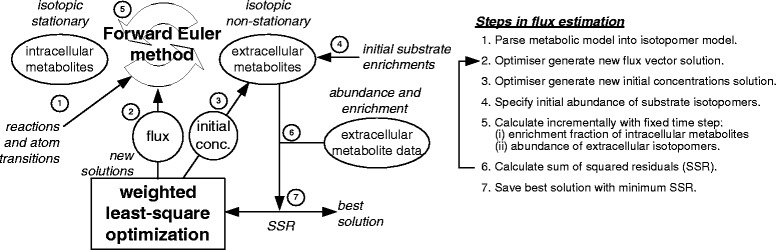
A hybrid ^13^C-MFA approach. Intracellular metabolites were assumed to turnover rapidly and thus achieve steady state enrichments, whereas large extracellular metabolite pools (lactate and pyruvate) accumulate over time and have transient enrichments. The forward (explicit) Euler method was used to perform the numerical integration using 1- and 2-min time steps to fit the HEK293 and PANC-1 data, respectively. Weighted least-square optimization was used to generate new flux and initial concentration estimates based on the difference between measured and simulated values

MATLAB R2012a (MathWorks, Natick, MA) was used for the following computation tasks: (1) GCMS peak integration, (2) correction for mass interference from non-carbon backbone isotopes [30], (3) enumeration of elementary modes, and (4) flux estimation by constraint-based least-square fitting of experimental data (Fig. 2). Tracer model was generated from the reaction network and atom transitions using OpenFLUX (see Additional file 1: S8) [31]. This research includes computations using the Linux computational cluster Katana supported by the Faculty of Science, UNSW Australia.

The simulation of isotopic non-stationary extracellular metabolite data was achieved using the forward Euler method (Fig. 2). It was assumed that cells are at metabolic steady state and intracellular metabolites are at isotopic steady state. The isotopic assumption was made on the basis that the turnover rates of intracellular metabolites are significantly faster compared to extracellular pyruvate and lactate. This assumption is reasonable because intracellular metabolite concentration range from 0.1 to 20 fmol/cell [32], which is three orders of magnitude smaller than the concentration range of extracellular pyruvate and lactate of 2 to 20 pmol/cell (2 ml culture, 1 million cells). Additionally, it has been observed that glycolytic metabolites can reach isotopic steady state within 1.5 h in CHO cells [25]. Hence, the model is a hybrid of isotopic non-stationary and isotopic stationary for extracellular and intracellular metabolites, respectively. During each time step, the steady-state intracellular enrichments are calculated based on extracellular enrichments; at the end of each time step, the extracellular enrichments (of pyruvate and lactate) are updated.

**Fig. 3:**
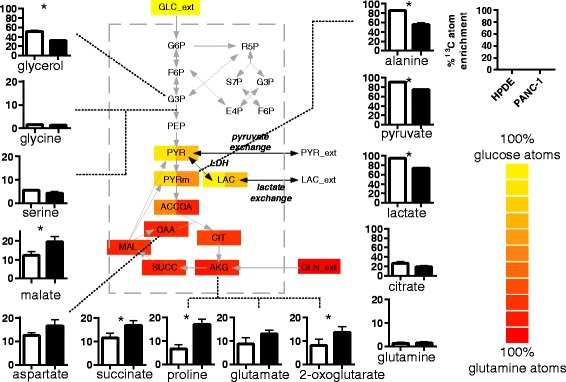
Enrichment of extracellular metabolites of HPDE and PANC-1 cells on DMEM labelled with [U-^13^C_6_]-glucose. *Bar graphs* show fractional labelling of metabolites measured in triplicates. Alanine enrichment was used as proxy for mitochondrial pyruvate. Acetyl-CoA enrichment was calculated from malate and citrate enrichment. Glucose carbon atoms appear to overflow into lactate, with a small portion entering TCA cycle. Heat map showed a greater dilution of glucose carbons (i.e., loss of ^13^C enrichment) in PANC-1 cells compared to HPDE cells. *Asterisk* indicates *p* < 0.05

Flux analysis was performed by least-square parameter estimation [33]. The same metabolic model was used in the flux analysis of both HEK and PANC-1 data (Fig. 3). An incentive of using forward Euler method is that the optimization problem is computationally less demanding to solve. Fixed time steps were arbitrarily set to 1 and 2 min for the HEK and PANC-1 data, which were sampled 30 and 60 min apart, respectively. The objective function minimised consists of the weighted differences of fraction enrichments and total abundances of metabolites between the measured and simulated values. The metabolites are lactate, pyruvate and glucose. For metabolite abundances, the error variances were calculated from their respective calibration curves. For fractional enrichments, a uniform error of 0.01 was assumed. This error was derived from the maximum discrepancy observed between the measured and the theoretical enrichments of natural lactate, pyruvate and glucose. Glucose showed the greatest source of error. To account for the inability to accurately specify the composition of glucose, we considered a more conservative approach of applying a uniform weighting error of 0.01 to calculate the sum of squared residuals, rather than using their actual measurement errors.

Sensitivity analysis was performed on the PANC-1 data by a Monte Carlo approach. The procedure resembles the experiments but performed in silico. Using the measured values and errors, 50 iterations of the experiment were performed by first “corrupting” the measured values with normally distributed errors and then running the optimization to estimate flux parameters. Flux standard errors were then calculated from the 50 sets of fluxes.

### Statistical analyses

Comparison of % ^13^C atom enrichments for PANC-1 and HPDE were expressed as means ± standard error of the mean (SEM). Results were analysed by unpaired Student’s *t* test between cell types assuming normal distribution and the same population standard deviations. Differences with *p* < 0.05 were deemed statistically significant.

## Results

### Extracellular TCA cycle metabolites enriched

The detection of ^13^C enriched central carbon metabolites in the culture medium of PANC-1 and HPDE cells fed with [U-^13^C_6_]-glucose for 24 h demonstrated extensive secretion of glucose-derived intracellular metabolites (Fig. 3). The % ^13^C atom enrichments, also known as fractional labelling [34] showed the fraction of carbon atoms of extracellular metabolites synthesised de novo from glucose. The enriched extracellular metabolites were not only conventional mammalian cell culture by-products like lactate, alanine and glutamate but also included TCA cycle metabolites and non-essential amino acids (e.g., proline and serine). Parallel unlabelled experiments were performed to establish baseline levels of natural enrichments, which were about 1–2 % ^13^C atom enrichment (see Additional file 1: S1). Among the measured metabolites, only glycine and glutamine were not enriched, indicating that PANC-1 and HPDE cells did not synthesise (and secrete) these metabolites from glucose.

Carbon atoms of the extracellular TCA cycle metabolites were up to 20 % labelled (Fig. 3). The measured enrichments were consistent with observations in similar PDAC cell lines that TCA cycle metabolite were predominantly derived from glutamine over glucose [13, 26]. Among the labelled fractions, the M2 fractions were the most pronounced, suggesting that TCA cycle was engaged canonically, i.e., oxidising acetyl-CoA. This was followed closely by the M3 fractions, which can be explained by anapleurotic flux, since neither the M3 nor M4 fraction of 2-oxoglutarate was higher than the M3 fractions of malate, succinate and aspartate (see Additional file 1: S2); 10 to 15 % glutamate atoms were derived from glucose atoms despite the backdrop of glutamine-driven anapleurotic flux. This suggested significant keto acid to amino acid inter-conversion by transaminases and was shown to occur in CHO cells as well (~15 % unlabelled glutamate when cells were cultured in uniformly labelled glutamine) [25].

We qualitatively assessed the metabolites present in DMEM and the initial cell culture medium (serum + DMEM). We found the serum to be the major contributor of unlabelled lactate and some of the amino acids and TCA cycle metabolites (Table 1). Dialysed serum did not contain any additional metabolites (results not shown) and thus should be considered as a substitute for serum [22]. In addition to being consumed, citrate and glycerol were enriched with glucose carbon atoms, evidence that these metabolites were exchanged between intracellular and extracellular pools.

**Table 1:** Metabolites detected in 10 μl cell culture medium measured by GC-MS

Metabolite	DMEM	DMEM and serum	HPDE cells	PDAC cells
Lactate	−	+	+++	+++
Pyruvate	+	+	+++	+++
Succinate	−	+	−	+
2-oxoglutarate	+	+	++	−
Malate	−	++	+	++
Citrate	−	+++	+	++
Glycerol	+	+++	+	++
Alanine	−	+	++	++
Aspartate	−	+	+	+
Glutamate	−	+	++	++
Glutamine	+	++	+	+
Glycine	+	+	+	++
Serine	+	+	+	+
Proline	−	+	+	++

Concentrations were scored qualitatively with “+”, “++” and “+++” to show relative quantity. Metabolites with peak area less than 5 % of the largest peak are marked with “−”

### Extracellular metabolites from PANC-1 and HPDE cells were differentially enriched

Sampled 24 h after swapping culture medium to the same DMEM containing [U-^13^C_6_] glucose, 75 ± 1 and 91 ± 1 % of extracellular pyruvate carbon atoms were found to be derived from glucose in PANC-1 and HPDE cells, respectively. The same trends were observed in the ^13^C enrichments for both alanine and lactate (Fig. 3). This suggested a greater contribution of unlabelled substrates to pyruvate production in PANC-1 cells.

The lower % ^13^C atom enrichment for alanine (56 ± 2 % for PANC-1, 85 ± 1 % for HPDE) compared to pyruvate suggested that mitochondrial pyruvate was less labelled than its cytosolic counterpart. This interpretation is drawn from the notion that mitochondrial alanine transaminase (ALAT 2) is the dominant enzyme in alanine production by cancer cells [23, 24] and that pancreatic tissue only expresses mitochondrial ALAT2 [35]. The dilution of alanine enrichment seen in PANC-1 compared to HPDE cells suggested a greater conversion of non-glucose carbon sources to mitochondria pyruvate in PANC-1 cells, e.g., glutaminolysis.

Surprisingly, TCA cycle metabolites were more enriched in PANC-1 than HPDE cells (Fig. 3). The M2 and M3 fractions were key contributors to the greater % ^13^C atom enrichment, pointing to a greater oxidation of pyruvate via pyruvate dehydrogenase and isocitrate dehydrogenase and by via anapleurotic pathway pyruvate carboxylase in PANC-1 cells. Citrate’s % ^13^C atom enrichment was greater in HPDE cells (26 ± 3 vs. 19 ± 1 %), but not significantly (*p* = 0.08), due to an unusually high M2 fraction (40–50 vs. 18 %), but the high M2 fraction did not propagate to 2-oxoglutarate and downstream metabolites. Put together, PANC-1 cells showed a greater dilution of pyruvate with unlabelled substrates compared to HPDE cells, but at the same time, a greater connectivity/mixing of pyruvate with TCA cycle intermediates.

### Buffering effect of a large extracellular lactate pool

In PANC-1 and HPDE cells, the enrichment of extracellular pyruvate mirrored that of lactate (Fig. 3), implying a strong connection between both metabolite pools. To test the hypothesis of a “leaky cell” involving rapid exchange fluxes around the pyruvate node, we assessed the enrichment rate of pyruvate of cells exposed to a low and a high lactate concentration. Without rapid exchanges, the enrichment of extracellular pyruvate should be proportional to glucose and independent of lactate enrichment.

**Fig. 4:**
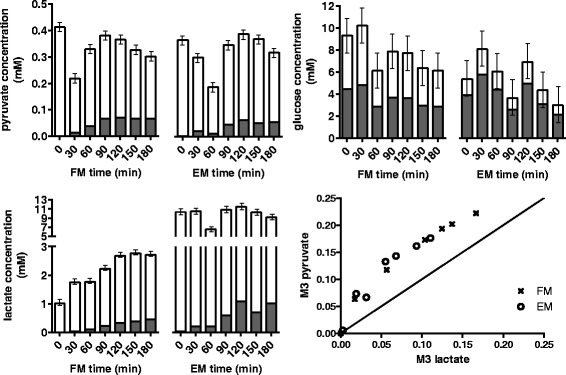
Concentration profiles of extracellular metabolites pyruvate, lactate and glucose from culture medium of HEK293 cells. Unlabelled and fully labelled fractions are shown as *white* and *grey bars*. Pyruvate, lactate and glucose concentrations were quantified using ions 142, 115 and 314, respectively. Pyruvate in EM was slightly less enriched than in FM despite glucose in EM was more labelled than in FM (71.7 vs. 46.9 %). Pyruvate is proximal to glucose compared to lactate and thus was more enriched. Pyruvate enrichment, however, correlated strongly with lactate’s

We conducted a 3-h time course of HEK293 cell cultures, measuring enrichment of extracellular pyruvate and lactate every half hour. Two experiments were set up: exponentially growing HEK cells were either refreshed to a new medium (experiment referred as FM) or cultured in the existing medium (experiment referred as EM), both with spike-in of 1 g/L [U-^13^C_6_]-glucose at experiment start (Fig. 4). The initial lactate concentrations of FM and EM cultures were about 1 and 10 mM, respectively. The labelled glucose fractions were at 46.9 % (FM) and 71.7 % (EM).

Except for the first time point, extracellular pyruvate in EM was slightly but consistently less enriched than in FM, despite EM having a greater fraction of labelled glucose (Fig. 4). The M3 enrichment fraction of lactate trailed behind pyruvate with an average gap of 6 %, for both FM and EM. The fraction of M3 increase was slower in EM because the culture had 10-fold more lactate than FM. More importantly, this buffering or dampening effect was extended to extracellular pyruvate, which was no longer strongly dependent on glucose enrichment. Thus, we inferred that both intracellular and extracellular pools of lactate and pyruvate pools can exchange reversibly and rapidly.

### Extracellular enrichments as proxies for intracellular pools

**Fig. 5:**
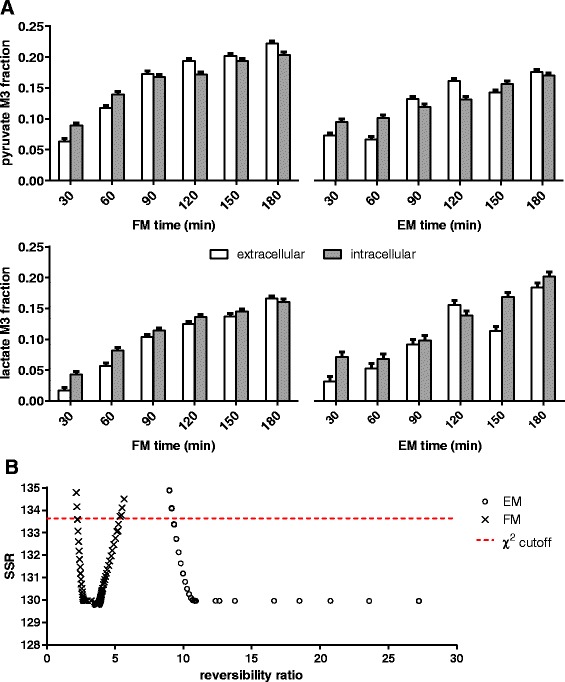
Comparing extracellular and intracellular M3 fraction of pyruvate and lactate. **a** Extracellular enrichments closely mirrored intracellular enrichments for both FM and EM experiments. Unlabelled lactate was present in the initial culture medium; thus, extracellular lactate was less enriched than intracellular lactate. The differences, however, were not inflated by a 10-fold larger extracellular pool size in EM (10 vs. 1 mM). **b** Reversibility ratios of lactate exchange and the calculated SSRs (residual sum of squares) for FM and EM experiments. *χ* ^2^(DoF = 1, *p* = 0.05) of 3.84 was used to determine the ratios’ 95 % confidence intervals. A reaction can be treated as fully reversible or irreversible if the calculated reversibility ratio is ≥25 or ≤0.05, respectively

Intracellular metabolites were also obtained for the FM and EM experiments to assess the extent of exchange between extracellular and intracellular pools. The M3 fractions of pyruvate and lactate were found to show similar trajectories and relative abundances compared to the intracellular counterparts over the 3-h time course (Fig. 5a). For FM, the deviations between intracellular and extracellular fractional enrichments were generally larger during the first hour. Paired two-tailed *t* test showed that intracellular lactate was consistently more enriched than the extracellular pool (*p* = 0.047), whereas no difference was found between the two pyruvate pools (*p* = 0.899). For EM, the intracellular and extracellular enrichments of lactate were similar (*p* = 0.119), with the intracellular pool consistently being slightly more enriched. There was also no obvious difference in enrichment between the two pyruvate pools under the EM condition (*p* = 0.707).

Overall, the results showed that extracellular and intracellular pyruvate were exchanging rapidly. The lactate results, however, showed a small enrichment lag, but interestingly, the differences in enrichment between extracellular and intracellular pools were not greater for EM despite having 10-fold more lactate than FM. The compensation may be have been achieved by a rate of exchange that is concentration dependent, i.e., faster exchange rate for higher concentrations.

### Lactate exchange was high, but not fully reversible

Flux analysis was used to characterise the limits of lactate exchange and to weigh-in on the importance of including this flux parameter in future tracer models. Particularly for such a nuisance parameter, whether by experimental design, there is an opportunity to render the dampening/buffering effect negligible and thus ignore the exchange flux by keeping the initial lactate concentrations low via medium refresh (e.g., FM). Otherwise, a direct spike-in (e.g., EM) approach is preferred because disturbances to the cells are minimised.

The reaction network model used for flux analysis consists of glycolysis, glutaminolysis, pentose-phosphate pathway (PPP) and TCA cycle (Fig. 3). The pools of labelled and unlabelled cytoplasmic pyruvate are mainly produced via glycolysis and glutaminolysis, respectively, and are consumed proportionally via the TCA cycle. The balance is accumulated reversibly as extracellular pyruvate and lactate.

The aim is to estimate the exchange fluxes of lactate using the abundance and enrichment of extracellular lactate, pyruvate and glucose. The magnitude of buffering lactate has on pyruvate is correlated to the lactate exchange flux and the extracellular lactate abundance. Here, our estimation approach will leverage the 10-fold contrast in extracellular lactate abundance (EM vs. FM) against the assumption of identical pyruvate producing and consuming fluxes between the parallel EM and FM experiments (apart from the lactate exchange flux). Note that when the EM and FM datasets were fitted separately, the ratios of glutaminolysis to glycolysis were found to be similar (see Additional file 1: S5). This confirmed that our assumption used for flux normalisation was reasonable and that an increased glutaminolysis could not explain the lower pyruvate enrichment observed for EM.

Here, we define the reversibility ratio of a reaction as a measure of the cyclic conversion between two metabolite pools relative to the net conversion of the reactant to the product [36]. The enrichment consistency between two mixing pools with respect to the reversibility ratio follows an asymptote. For a simple two-pool mixing problem, e.g., *x*/(*x* + 1), reversibility ratios of 0.1, 1, 2, 10 and 100 will yield 9.1, 50, 66.7, 91 and 99 % of the equilibrium limit. Given a 5 % margin of error, a reaction can be consider fully reversible when the reversibility ratio is ≥20 or irreversible when the reversibility ratio is ≤0.05.

Flux analysis gave optimum reversibility ratios of 4 and 312 for FM and EM, respectively. By constraining the reversibility ratios to be the same, the residual error increased from 130 to 165, giving a combined optimum reversibility ratio of 94. Different lactate exchange fluxes for FM and EM were therefore required to produce a better fit. The lower limit of the reversibility ratios were subsequently determined by allowing the best residual error to increase by 3.84 (*χ*^2^_0.05,1_) [33]. The lower limits were 2.3 and 9.2 for FM and EM, respectively, indicating significant lactate exchange flux, even for FM that had a lower lactate concentration (Fig. 5b). With the same approach, the upper limit for FM was estimated to be 5.2, indicating that lactate exchange cannot be assigned as fully reversible by-default in a medium with low lactate.

The calculated lower limits (>2.3) strongly suggested that lactate exchange must be treated as a bi-directional flux in ^13^C-MFA, particularly when the initial concentration of extracellular lactate is 1 mM or greater. Unless a lactate-free medium is used (e.g., serum-free or dialysed serum), the exchange of unlabelled extracellular lactate will cause the dilution of pyruvate enrichment. Flux results also supported the possibility that the rate of lactate exchange was concentration dependent. Lactate exchange was not fully reversible when extracellular lactate was low, as such, one also cannot simplify the ^13^C-MFA model by assuming a fully reversible lactate exchange. Ultimately, the modelling treatment of lactate exchange must be evaluated on a case-by-case basis.

### PANC-1 cells treated with TNF-α increased fluxes into pyruvate

We have established that ^13^C enrichment of extracellular pyruvate is a good proxy for intracellular pool and that lactate exchange is significant and bi-directional. We followed to apply our findings in quantifying metabolic differences in PANC-1 cells treated with TNF-α by ^13^C flux analysis, using only abundances and enrichments of lactate, pyruvate and glucose. The activity of glycolysis and glutaminolysis can be inferred from the ^13^C enrichment pattern and abundance of pyruvate and lactate (see Additional file 1: S6). Apart from evidence that it promotes aerobic glycolysis [37], there is relatively limited knowledge about the metabolic effects of TNF-α.

**Fig. 6:**
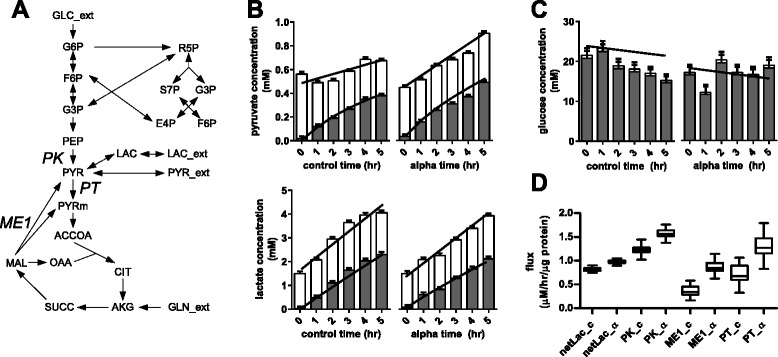
Extracellular metabolites and flux changes in PANC-1 cells treated with TNF-α. *Bars* show abundance of unlabelled (*white*) and fully labelled (*grey*) fractions. *Trend lines* show fitting of simulated data to experimental data generated by constrained non-linear least-square regression. **a** Metabolic model used to simulate data to fit experimental data. The model included glycolysis, glutaminolysis, PPP, TCA cycle, and the reversible exchange of lactate and pyruvate between intracellular and extracellular pools (see Additional file 1: S8). **b** Concentration profiles of extracellular pyruvate and lactate. **c** Concentration profile of glucose. **d** Estimated fluxes showing metabolic differences in PANC-1 cells with and without TNF-α as treatment. Flux intervals were generated by a Monte Carlo approach. *netLac* net lactate efflux, *PK* pyruvate kinase, *ME1* cytoplasmic NADP-dependent malic enzyme, *PT* mitochondrial pyruvate carrier, *_c* control, *_α* TNF-α

The same reaction network model as HEK293 was used (Fig. 6a). The aim of this flux analysis was to quantify fluxes of pathways producing and consuming pyruvate using extracellular metabolites.

Several metabolic pathways were excluded due to lack of data. Here, we used glutamine as a generic source of substrates not derived from glucose, lactate and pyruvate. This includes the uptake of unlabelled TCA cycle intermediates from the medium and the catabolism of amino acids into unlabelled cytoplasmic pyruvate. They were lumped into the flux contribution of cytoplasmic malic enzyme (ME1). Except for serine and glycine, the catabolic pathways of all other amino acids and fatty acids will only feed into pyruvate and TCA intermediates (acetyl-CoA, α-ketoglutarate, succinate, fumarate and oxaloacetate). When labelled glucose is used, enrichment data cannot differentially resolve the activity of these pathways from glutaminolysis, all of which contribute unlabelled carbon to the system. Pyruvate carboxylase was omitted because anapleurotic flux cannot be resolved from glutaminolysis without the enrichment data of TCA cycle intermediates. While alanine and aspartate are known to be significant by-products, it was assumed that their secretion rates were small compared to lactate. It was also assumed that biomass drains were negligible compared to the fluxes described in the model, especially when the cell culture experiments were kept short (≤5 h).

For both control and TNF-α-treated cells, extracellular enrichment data showed accumulation of the M3 fraction of lactate and pyruvate, but no appreciable accumulation of unlabelled metabolites (Fig. 6b). Having established the precedence of significant lactate exchange, we no longer interpret this observation as “lactate being solely produced from glucose”. Corresponding intracellular data showed that cells had almost 60 % unlabelled pyruvate (see Additional file 1: S7), reinforcing the entry of lactate and other unlabelled substrates.

Flux results showed that the net lactate secretion rates had increased from 0.81 to 0.97 μM/h/μg protein with TNF-α treatment (*p* < 0.001) (Fig. 6b). There was, however, a greater increase in the ME1 flux (from 0.36 to 0.86 ± 0.02 μM/h/μg protein) compared to the glycolysis flux at pyruvate kinase (PK) (from 1.23 to 1.58 ± 0.01 μM/h/μg protein). The net flux of mitochondrial pyruvate carrier (PT) increased by 0.58 ± 0.04 μM/h/μg protein, suggesting that the oxidation of pyruvate via the TCA cycle was increased in concomitant with a greater pyruvate influx. The balance was secreted directly as pyruvate, indicated by the increased accumulation rate of extracellular pyruvate from 0.05 to 0.16 μM/h/μg protein.

Overall, flux results suggested that TNF-α treatment of PANC-1 cells not only caused the up-regulation of glycolysis but also increased fluxes toward pyruvate and the oxidation of pyruvate via the TCA cycle. Note that the ME1 flux integrates unspecified sources of unlabelled cytoplasmic pyruvate by lumping them together with glutaminolysis. This draws away potential interference that will alter estimates for PK, PT and the extracellular accumulation fluxes. Also, the exclusion of potential effluxes for TCA cycle metabolites does not suppress the flux through PT because the TCA cycle can freely oxidise pyruvate via the mitochondrial malic enzyme. Altogether, the fluxes estimated around the pyruvate node (Fig. 6d) will remain the same with or without the free exchange of TCA cycle metabolites.

## Discussion

This work demonstrated significant exchange of pyruvate and lactate between extracellular and intracellular pools, which affected the enrichment of pyruvate and downstream metabolites. Intracellular pyruvate enrichment is therefore a function of extracellular lactate and pyruvate enrichment, not just incoming glucose and glutamine. The large extracellular lactate pool appeared to partially explain the dampened dynamics seen in pyruvate enrichment compared to other glycolytic intermediates in a labelled glucose experiment. Although medium refresh is recommended to minimise interference caused by metabolic exchange of metabolites, including lactate [34], the use of serum makes it impossible to completely eliminate these issues. Unless defined medium or dialysed serum is used when performing ^13^C-MFA, flux analysis should account for a reversible lactate exchange. The absence of large initial extracellular lactate will significantly shorten the time required to achieve isotopic steady state for pyruvate and downstream metabolites. The results from the current study indicates that because of an apparent free exchange between pools, when performing ^13^C flux analysis in cultured cells, extracellular enrichment data should be considered, in addition to intracellular data.

There were two main tracer findings in the present work that confirmed a fast lactate exchange flux. Firstly, both extracellular pyruvate and lactate enrichment fractions showed similar trajectories. Although the direct inter-conversion of extracellular pyruvate and lactate could explain this observation, this phenomenon is very unlikely to occur. Intracellular pyruvate and lactate enrichments, which also showed similar trajectories, were key to confirm that the intracellular route was taken to convert lactate to pyruvate and vice-versa. The second evidence was that more labelled glucose did not lead to more labelled pyruvate. Phosphoenolpyruvate, which mirrors glucose enrichment, was essentially feeding into a large pyruvate node that includes both extracellular and intracellular pyruvate and lactate. The rate of intracellular pyruvate enrichment is therefore dependent on the abundance of extracellular lactate and pyruvate. The flux modelling performed is a form of hypothesis testing, whereby a reversible lactate exchange, i.e., a missing reaction [38], must be provided to adequately fit the FM and EM enrichment data.

There is a growing recognition that lactate is both a substrate as well a by-product. Literature has suggested that tumours can utilise lactate [10], and this phenomenon is often referred to by different terms, such as metabolic symbiosis [39], two-compartment nutrient-sharing model [2], reverse Warburg effect [18], and tumour-stroma co-evolution [40, 41]. Cells switched to lactate consumption upon glucose depletion without any appreciable metabolic adjustment [42], and lactate can contribute to the by-products alanine and glutamate in mammary carcinomas even in the presence of glucose [43]. Tumours are known for their ability to scavenge nutrients, such as fatty acids, branched-chain amino acids and acetate [1, 20]; pyruvate serves as an even more versatile energy and biomass precursor. Here, we showed that cells essentially have a large pyruvate pool that extends beyond the cell membrane. Rather than being deliberately up-regulated, the tumour cells’ ability to dynamically access extracellular lactate to refill intracellular pyruvate may a persistent background process.

Glutamine, catabolised via the TCA cycle, has a very diverse contribution to energy, biosynthesis and redox balance [44]. By tracer experiments, the nutrient has been linked to a compensatory role when mitochondrial pyruvate carrier or pyruvate dehydrogenase was suppressed [14, 23, 24], to the increased invasiveness of ovarian cancer [12], and to lipogenesis in hypoxic condition [45]. As it appears that careful maintenance of TCA metabolite pools would be crucial to cell proliferation, the detection of 10–20 % enrichment in TCA cycle intermediates in a labelled glucose experiment—extracellularly—was therefore unexpected. This means that, for the aforementioned labelled glutamine experiments, if labelled metabolites are lost to the medium, then the flux of labelled metabolites will taper off with increasing distance from 2-oxoglutarate due to leakages. The anapleurosis role of glutamine is therefore even more pervasive than the current estimates if compensation for these losses is necessary. However, there may be other substrates that can refill TCA cycle metabolites. For example, in the control C2C12 myoblasts, TCA cycle metabolites succinate, fumarate, malate and 2-oxoglutarate were in total 55 to 70 % labelled by both glucose and glutamine [24], with the balance unaccounted for. We raised the exchange issue regarding TCA cycle metabolites with the HPDE vs. PANC-1 experiment, where enriched TCA cycle metabolites were found not only in the medium but were also differentially enriched between the normal and cancerous cell lines. More data, however, will be required to quantify individual metabolites’ exchange dynamics.

The “leaky” cell model can be used to our advantage to continuously monitor metabolism without harvesting cells, simply by collecting small amounts of culture medium over time. We showed that extracellular pyruvate and lactate closely mirrored intracellular enrichments, albeit the latter displayed a slight lag. Under this framework, TNF-α was used as a model to investigate effects of chronic inflammation on cancer progression and the metabolic reprogramming involved in metastasis. With ^13^C enrichment and abundance of glucose, lactate and pyruvate, PANC-1 cells treated with TNF-α were shown to have increased glycolysis and lactate flux, as well as the conversion of non-glucose substrates to pyruvate represented by ME1 flux. Note that the increase in pyruvate oxidation was inferred by flux balancing (i.e., lactate and pyruvate net production rates were constrained) and therefore will require further validation. In prostate and breast epithelial cells, TNF-α was shown to increase aerobic glycolysis but not the TCA cycle activity [37, 46].

Tracer experiments are very effective grounds for testing metabolic hypothesis, particularly in light of the near-boundless design space and the ever-expanding analytical coverage [47–49]. Parameter identifiability is affected by the input substrate specification, and therefore identifying the range of impinging substrates can broaden our design scope. For example, this study suggests the possibility of using labelled lactate to quantify TCA cycle fluxes even if the cell is actively producing lactate.

## Conclusions

While tracer experiments provide information on the sources that have contributed to the synthesis of a particular metabolite, metabolic activity is inferred and not measured directly. Tracer data therefore must be interpreted in the right context. Here, we raised the prospect that lactate is a significant input substrate in tracer experiments if the metabolite is present in the medium. This finding adequately explains why the enrichment of pyruvate sustains such a long lag in a labelled glucose experiment. This issue of significant exchange flux is also applicable to TCA cycle metabolites and potentially others as well. Nonetheless, the exchange fluxes between intracellular and extracellular pools present an opportunity to profile metabolism in a time-resolved manner, simply by sampling the culture medium.

## Additional file

Additional file 1: S1. Baseline enrichment of unlabelled experiment and comparison to labelled experiment. **S2.** GC-MS peaks from DMEM media with and without serum and from HPDE/PDAC cell culture medium. **S3.** GC-MS chromatogram of low glucose DMEM derivatised by MOX-TMS. **S4.** Methoximation-acetylation-butylation (MAB) derivatization. **S5.** Flux analysis for FM and EM experiments. **S6.** Pathway carbon atom mapping. **S7.** Intracellular enrichment of PANC-1 cells at 10 hour. **S8.** OpenFLUX model for HEK 293 and PANC-1 cells. (DOCX 3503 kb)
