# Supplementary material for: Fast exchange fluxes around the pyruvate node: a leaky cell model to explain the gain and loss of unlabelled and labelled metabolites in a tracer experiment
Source: Cancer Metab. 2016 Jul 4;4:13. doi: 10.1186/s40170-016-0153-9 (PMC4931697; doi:10.1186/s40170-016-0153-9)
Supplement: Additional file 1: S1. — Baseline enrichment of unlabelled experiment and comparison to labelled experiment. S2. GC-MS peaks from DMEM media with and without serum and from HPDE/PDAC cell culture medium. S3. GC-MS chromatogram of low glucose DMEM derivatised by MOX-TMS. S4. Methoximation-acetylation-butylation (MAB) derivatization. S5. Flux analysis for FM and EM experiments. S6. Pathway carbon atom mapping. S7. Intracellular enrichment of PANC-1 cells at 10 hour. S8. OpenFLUX model for HEK 293 and PANC-1 cells. (DOCX 3503 kb) [file 40170_2016_153_MOESM1_ESM.docx]

Fast exchange fluxes around the pyruvate node: a leaky cell model to explain the gain and loss of unlabelled and labelled metabolites in a tracer experiment

Lake-Ee Quek, Menghan Liu, Sanket Joshi, Nigel Turner

Table of content

S1. Baseline enrichment of unlabelled experiment and comparison to labelled experiment

S2. GCMS peaks of metabolites from DMEM media with and without serum, and from HPDE/PDAC cell culture medium

S3. GCMS chromatogram of low glucose DMEM derivatized by MOX-TMS

S4. Methoximation-acetylation-butylation derivatization

S5. Flux analysis of FM and EM experiments

S6. Pathway carbon atom mapping

S7. Intracellular enrichment of PANC-1 cells at 10 hour

S8. OpenFLUX model for HEK 293 and PANC-1 cells

S1. Baseline enrichment of unlabelled experiment and comparison to labelled experiment

PDAC and HPDE cell cultures were performed in triplicates in normal medium and in medium with 100% [U-13C]-glucose for 24 hours. Mass isotopomer distributions of extracellular metabolites were obtained by SIM (selective ion monitoring) mode, or by scan mode if SIM windows were not specified. Mass distributions shown in the table below have been corrected for interference from natural enrichment of non-carbon backbone atoms.

|  |  | PDAC |  |  |  |  |  |  | HPDE |  |  |  |  |  |
| --- | --- | --- | --- | --- | --- | --- | --- | --- | --- | --- | --- | --- | --- | --- |
|  |  | natural |  |  | labelled |  |  |  | natural |  |  | labelled |  |  |
|  |  | A | B | C | A | B | C |  | A | B | C | A | B | C |
| pyruvate | m0 | 0.97 | 0.969 | 0.968 | 0.233 | 0.225 | 0.247 |  | 0.97 | 0.971 | 0.971 | 0.083 | 0.095 | 0.072 |
|  | m1 | 0.03 | 0.031 | 0.032 | 0.015 | 0.015 | 0.017 |  | 0.03 | 0.029 | 0.029 | 0.004 | 0.004 | 0.004 |
|  | m2 | 0 | 0.001 | 0 | 0.018 | 0.019 | 0.02 |  | 0 | 0 | 0 | 0.012 | 0.012 | 0.013 |
|  | m3 | 0 | 0 | 0 | 0.733 | 0.741 | 0.716 |  | 0 | 0 | 0 | 0.901 | 0.889 | 0.91 |
|  |  |  |  |  |  |  |  |  |  |  |  |  |  |  |
| lactate | m0 | 0.969 | 0.977 | 0.974 | 0.241 | 0.227 | 0.256 |  | 0.976 | 0.973 | 0.972 | 0.047 | 0.043 | 0.046 |
|  | m1 | 0.029 | 0.023 | 0.026 | 0.015 | 0.015 | 0.017 |  | 0.024 | 0.027 | 0.028 | 0.003 | 0.003 | 0.004 |
|  | m2 | 0.002 | 0 | 0 | 0.02 | 0.02 | 0.021 |  | 0 | 0 | 0 | 0.014 | 0.014 | 0.015 |
|  | m3 | 0 | 0 | 0 | 0.724 | 0.738 | 0.706 |  | 0 | 0 | 0 | 0.935 | 0.939 | 0.935 |
|  |  |  |  |  |  |  |  |  |  |  |  |  |  |  |
| alanine | m0 | 0.974 | 0.973 | 0.975 | 0.414 | 0.398 | 0.46 |  | 0.97 | 0.971 | 0.97 | 0.144 | 0.145 | 0.125 |
|  | m1 | 0.026 | 0.027 | 0.025 | 0.019 | 0.019 | 0.021 |  | 0.03 | 0.029 | 0.03 | 0.008 | 0.009 | 0.009 |
|  | m2 | 0 | 0 | 0 | 0.014 | 0.015 | 0.015 |  | 0 | 0 | 0 | 0.014 | 0.016 | 0.017 |
|  | m3 | 0 | 0 | 0 | 0.553 | 0.569 | 0.504 |  | 0 | 0 | 0 | 0.833 | 0.831 | 0.849 |
|  |  |  |  |  |  |  |  |  |  |  |  |  |  |  |
| succinate | m0 | 0.953 | 0.953 | 0.953 | 0.71 | 0.705 | 0.65 |  | 0.881 | 0.897 | 0.88 | 0.792 | 0.781 | 0.729 |
|  | m1 | 0.043 | 0.044 | 0.044 | 0.081 | 0.077 | 0.088 |  | 0.088 | 0.086 | 0.097 | 0.101 | 0.078 | 0.088 |
|  | m2 | 0.002 | 0.002 | 0.002 | 0.124 | 0.126 | 0.148 |  | 0.014 | 0.006 | 0.009 | 0.069 | 0.084 | 0.122 |
|  | m3 | 0.001 | 0.001 | 0 | 0.06 | 0.064 | 0.073 |  | 0.008 | 0.005 | 0.007 | 0.016 | 0.024 | 0.035 |
|  | m4 | 0.001 | 0.001 | 0 | 0.027 | 0.029 | 0.041 |  | 0.009 | 0.006 | 0.007 | 0.022 | 0.033 | 0.026 |
|  |  |  |  |  |  |  |  |  |  |  |  |  |  |  |
| glycerol | m0 | 0.974 | 0.976 | 0.977 | 0.664 | 0.645 | 0.686 |  | 0.965 | 0.969 | 0.977 | 0.447 | 0.492 | 0.466 |
|  | m1 | 0.026 | 0.024 | 0.023 | 0.025 | 0.025 | 0.026 |  | 0.035 | 0.031 | 0.023 | 0.021 | 0.014 | 0.02 |
|  | m2 | 0 | 0 | 0 | 0.003 | 0.003 | 0.002 |  | 0 | 0 | 0 | 0.002 | 0 | 0 |
|  | m3 | 0 | 0 | 0 | 0.308 | 0.327 | 0.287 |  | 0 | 0 | 0 | 0.531 | 0.494 | 0.513 |
|  |  |  |  |  |  |  |  |  |  |  |  |  |  |  |
| 2-oxoglutarate | m0 | 0.95 | 0.949 | 0.949 | 0.74 | 0.734 | 0.677 |  | 0.959 | 0.956 | 0.958 | 0.845 | 0.822 | 0.752 |
|  | m1 | 0.05 | 0.051 | 0.051 | 0.058 | 0.059 | 0.06 |  | 0.041 | 0.044 | 0.042 | 0.044 | 0.038 | 0.042 |
|  | m2 | 0 | 0 | 0 | 0.117 | 0.12 | 0.136 |  | 0 | 0 | 0 | 0.095 | 0.114 | 0.151 |
|  | m3 | 0 | 0 | 0 | 0.038 | 0.039 | 0.053 |  | 0 | 0 | 0 | 0.006 | 0.011 | 0.024 |
|  | m4 | 0 | 0 | 0 | 0.031 | 0.032 | 0.047 |  | 0 | 0 | 0 | 0.007 | 0.011 | 0.024 |
|  | m5 | 0 | 0 | 0 | 0.016 | 0.017 | 0.027 |  | 0 | 0 | 0 | 0.002 | 0.004 | 0.008 |
|  |  |  |  |  |  |  |  |  |  |  |  |  |  |  |
| malate | m0 | 0.961 | 0.962 | 0.961 | 0.688 | 0.686 | 0.613 |  | 0.968 | 0.965 | 0.972 | 0.824 | 0.793 | 0.743 |
|  | m1 | 0.039 | 0.038 | 0.039 | 0.072 | 0.068 | 0.074 |  | 0.032 | 0.035 | 0.028 | 0.031 | 0.044 | 0.04 |
|  | m2 | 0 | 0 | 0 | 0.119 | 0.12 | 0.142 |  | 0 | 0 | 0 | 0.059 | 0.077 | 0.123 |
|  | m3 | 0 | 0 | 0 | 0.094 | 0.099 | 0.129 |  | 0 | 0 | 0 | 0.079 | 0.076 | 0.081 |
|  | m4 | 0 | 0 | 0 | 0.027 | 0.027 | 0.042 |  | 0 | 0 | 0 | 0.007 | 0.01 | 0.013 |
|  |  |  |  |  |  |  |  |  |  |  |  |  |  |  |
| aspartate | m0 | 0.963 | 0.963 | 0.964 | 0.733 | 0.725 | 0.662 |  | 0.974 | 0.974 | 0.977 | 0.819 | 0.806 | 0.751 |
|  | m1 | 0.037 | 0.037 | 0.036 | 0.066 | 0.067 | 0.071 |  | 0.026 | 0.026 | 0.023 | 0.029 | 0.034 | 0.039 |
|  | m2 | 0 | 0 | 0 | 0.098 | 0.103 | 0.124 |  | 0 | 0 | 0 | 0.058 | 0.074 | 0.125 |
|  | m3 | 0 | 0 | 0 | 0.081 | 0.084 | 0.108 |  | 0 | 0 | 0 | 0.056 | 0.057 | 0.066 |
|  | m4 | 0 | 0 | 0 | 0.022 | 0.022 | 0.035 |  | 0 | 0 | 0 | 0.038 | 0.028 | 0.018 |
|  |  |  |  |  |  |  |  |  |  |  |  |  |  |  |
| glutamate | m0 | 0.955 | 0.954 | 0.955 | 0.744 | 0.745 | 0.699 |  | 0.948 | 0.952 | 0.948 | 0.839 | 0.805 | 0.737 |
|  | m1 | 0.042 | 0.045 | 0.044 | 0.058 | 0.054 | 0.059 |  | 0.048 | 0.045 | 0.05 | 0.042 | 0.048 | 0.044 |
|  | m2 | 0.002 | 0 | 0.001 | 0.117 | 0.117 | 0.129 |  | 0.004 | 0.003 | 0.002 | 0.095 | 0.114 | 0.16 |
|  | m3 | 0 | 0 | 0 | 0.037 | 0.038 | 0.048 |  | 0 | 0 | 0 | 0.01 | 0.014 | 0.025 |
|  | m4 | 0 | 0 | 0 | 0.03 | 0.03 | 0.04 |  | 0 | 0 | 0 | 0.01 | 0.013 | 0.026 |
|  | m5 | 0 | 0 | 0 | 0.015 | 0.016 | 0.024 |  | 0 | 0 | 0 | 0.004 | 0.005 | 0.008 |
|  |  |  |  |  |  |  |  |  |  |  |  |  |  |  |
| citrate | m0 | 0.938 | 0.935 | 0.933 | 0.595 | 0.586 | 0.548 |  | 0.88 | 0.896 | 0.889 | 0.443 | 0.38 | 0.266 |
|  | m1 | 0.062 | 0.061 | 0.064 | 0.061 | 0.06 | 0.06 |  | 0.113 | 0.098 | 0.107 | 0.04 | 0.034 | 0.03 |
|  | m2 | 0 | 0.004 | 0.002 | 0.177 | 0.185 | 0.182 |  | 0.004 | 0.004 | 0.003 | 0.394 | 0.442 | 0.501 |
|  | m3 | 0 | 0 | 0 | 0.058 | 0.056 | 0.07 |  | 0.002 | 0.001 | 0 | 0.043 | 0.047 | 0.051 |
|  | m4 | 0 | 0 | 0 | 0.058 | 0.062 | 0.073 |  | 0.001 | 0 | 0 | 0.045 | 0.06 | 0.096 |
|  | m5 | 0 | 0 | 0 | 0.039 | 0.04 | 0.051 |  | 0 | 0 | 0 | 0.032 | 0.033 | 0.048 |
|  | m6 | 0 | 0 | 0 | 0.011 | 0.012 | 0.016 |  | 0 | 0 | 0.001 | 0.003 | 0.004 | 0.008 |
|  |  |  |  |  |  |  |  |  |  |  |  |  |  |  |
| glycine | m0 | 0.99 | 0.988 | 0.989 | 0.979 | 0.974 | 0.982 |  | 0.985 | 0.985 | 0.985 | 0.98 | 0.973 | 0.977 |
|  | m1 | 0.01 | 0.012 | 0.011 | 0.014 | 0.017 | 0.014 |  | 0.015 | 0.015 | 0.015 | 0.015 | 0.017 | 0.016 |
|  | m2 | 0 | 0 | 0 | 0.007 | 0.009 | 0.004 |  | 0 | 0 | 0 | 0.005 | 0.01 | 0.007 |
|  |  |  |  |  |  |  |  |  |  |  |  |  |  |  |
| proline | m0 | 0.955 | 0.95 | 0.952 | 0.714 | 0.705 | 0.656 |  | 0.934 | 0.943 | 0.952 | 0.884 | 0.86 | 0.834 |
|  | m1 | 0.045 | 0.045 | 0.046 | 0.077 | 0.082 | 0.081 |  | 0.054 | 0.042 | 0.026 | 0.062 | 0.062 | 0.036 |
|  | m2 | 0 | 0.001 | 0.001 | 0.116 | 0.123 | 0.145 |  | 0.012 | 0.012 | 0.015 | 0.035 | 0.068 | 0.091 |
|  | m3 | 0 | 0.001 | 0 | 0.057 | 0.055 | 0.068 |  | 0 | 0 | 0.004 | 0.015 | 0 | 0.028 |
|  | m4 | 0.001 | 0.002 | 0.001 | 0.035 | 0.037 | 0.051 |  | 0 | 0.003 | 0.002 | 0.005 | 0.009 | 0.012 |
|  |  |  |  |  |  |  |  |  |  |  |  |  |  |  |
| serine | m0 | 0.975 | 0.973 | 0.974 | 0.922 | 0.919 | 0.939 |  | 0.974 | 0.975 | 0.973 | 0.922 | 0.912 | 0.912 |
|  | m1 | 0.025 | 0.026 | 0.026 | 0.043 | 0.044 | 0.038 |  | 0.026 | 0.025 | 0.027 | 0.035 | 0.042 | 0.042 |
|  | m2 | 0 | 0 | 0 | 0.009 | 0.008 | 0.006 |  | 0 | 0 | 0 | 0.005 | 0.006 | 0.007 |
|  | m3 | 0 | 0 | 0 | 0.026 | 0.029 | 0.017 |  | 0 | 0 | 0 | 0.037 | 0.04 | 0.039 |
|  |  |  |  |  |  |  |  |  |  |  |  |  |  |  |
| glutamine | m0 | 0.952 | 0.898 | 0.945 | 0.948 | 0.95 | 0.898 |  | 0.945 | 0.948 | 0.947 | 0.945 | 0.954 | 0.947 |
|  | m1 | 0.048 | 0.099 | 0.052 | 0.05 | 0.047 | 0.095 |  | 0.051 | 0.05 | 0.049 | 0.05 | 0 | 0.048 |
|  | m2 | 0 | 0.003 | 0.003 | 0 | 0 | 0.006 |  | 0.002 | 0.001 | 0.004 | 0.005 | 0.046 | 0.004 |
|  | m3 | 0 | 0 | 0 | 0.001 | 0.002 | 0 |  | 0.002 | 0 | 0 | 0 | 0 | 0 |
|  | m4 | 0 | 0 | 0 | 0.001 | 0.001 | 0 |  | 0 | 0 | 0 | 0 | 0 | 0 |
|  |  | 0 | 0 | 0 | 0 | 0 | 0 |  | 0 | 0 | 0 | 0 | 0 | 0 |

S2. GCMS peaks of metabolites from DMEM media with and without serum, and from HPDE/PDAC cell culture medium

Screenshots of raw GCMS chromatograms obtained showing M-57 ion of metabolites. Peaks from DMEM + serum and DMEM samples appear to elute earlier because they were analysed on a shorter GC column.

black: DMEM + 10% FBS; blue: DMEM;

red: HPDE cell culture medium sample A 24 hr; green: PDAC cell culture medium sample A 24 hr

| Pyruvate 174  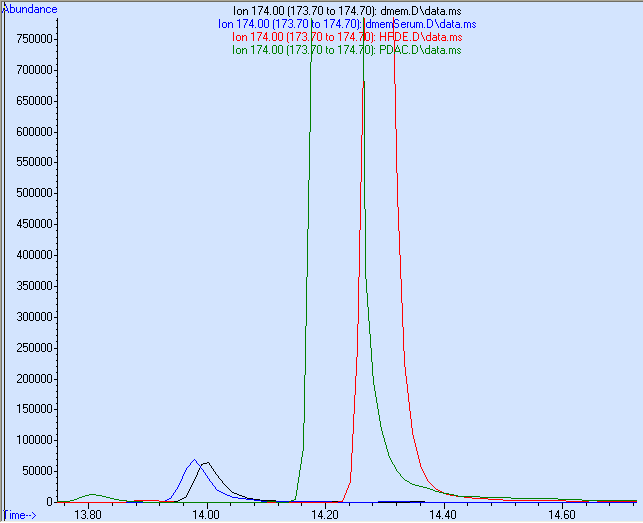  *very small peak from DMEM and serum  *significant production from cells | Lactate 261  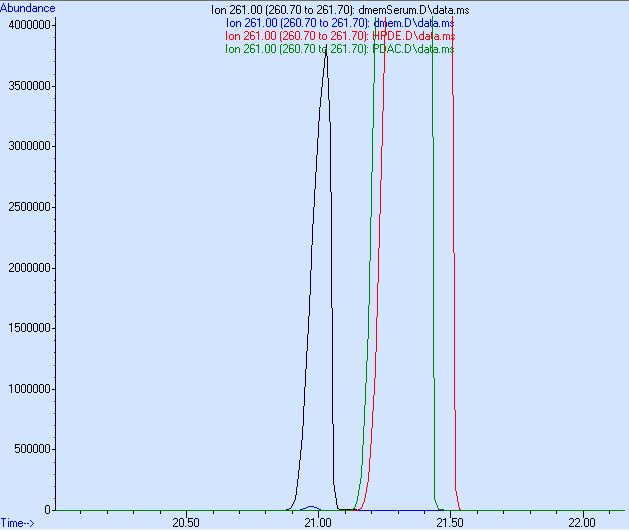  *absent in DMEM, present in serum  *significant production from cells |
| --- | --- |
| Alanine 260  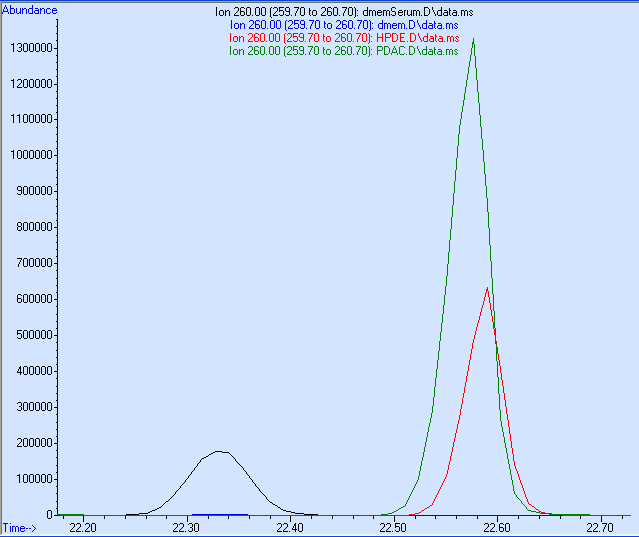  *absent in DMEM, present in serum  *produced | Glycine 246  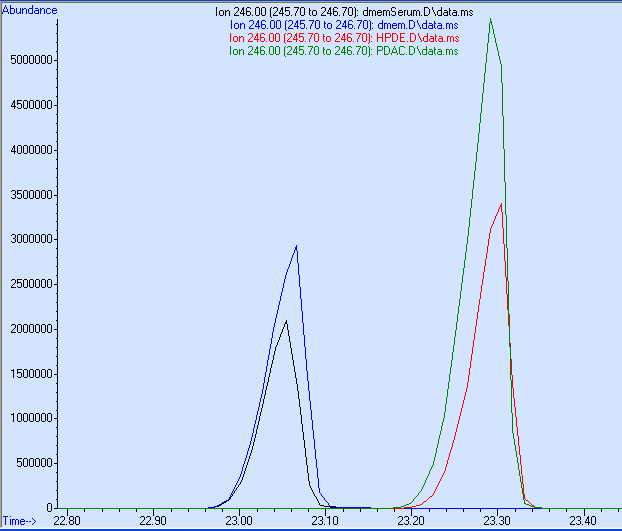  *present in DMEM  *produced |
| Succinate 289  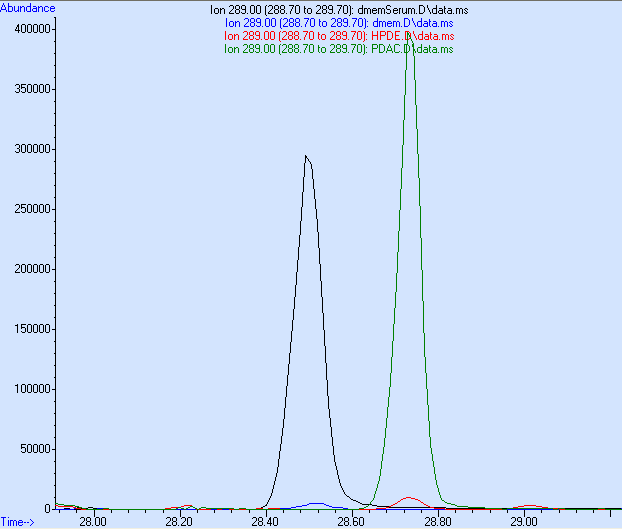  * absent in DMEM, present in serum  *produced and consumed | Proline 258  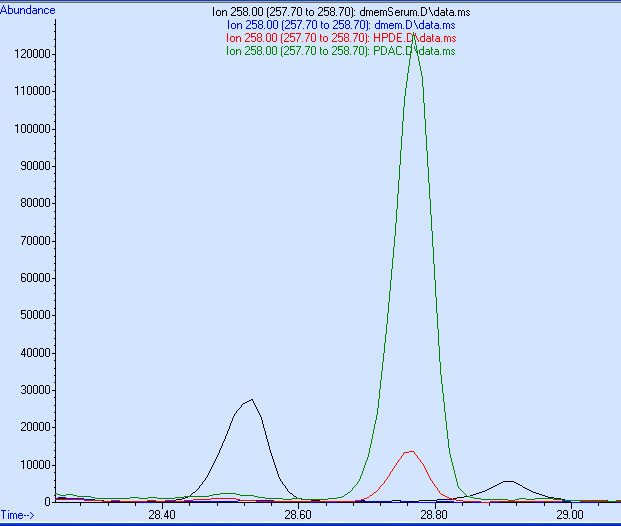  *proline elutes together with succinate, use M-285 instead  *absent in DMEM, present in serum  *produced |
| Glycerol 377  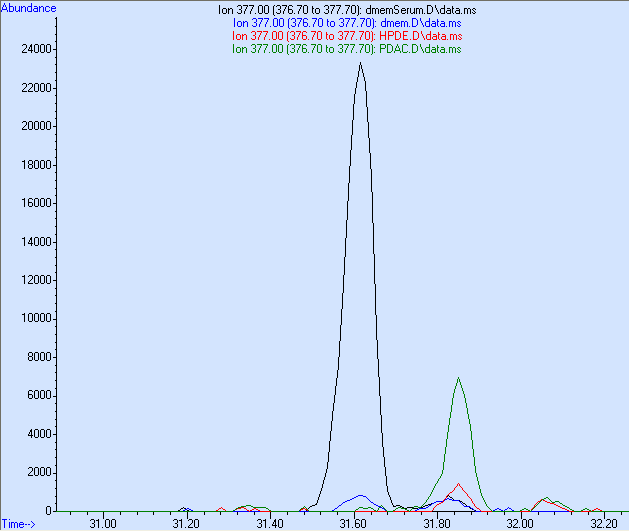  *absent in DMEM, present in serum  *consumed | Serine 390  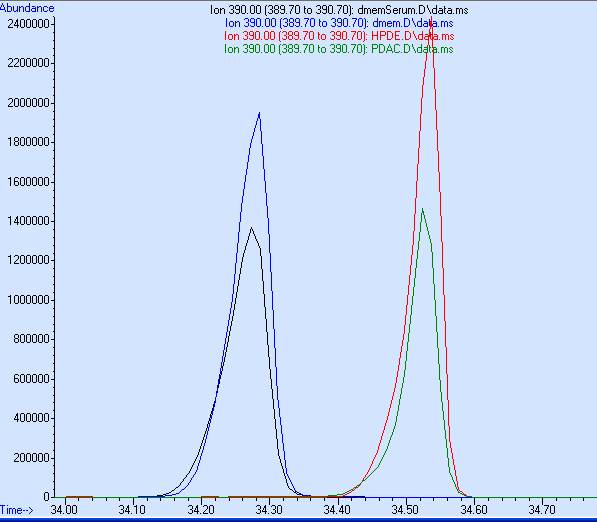  *present in DMEM |
| 2-oxoglutarate 346  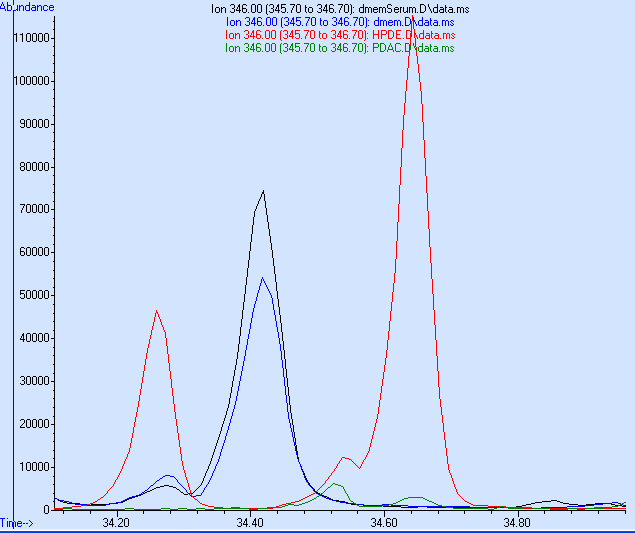  *present in DMEM  *produced and consumed | Malate 419  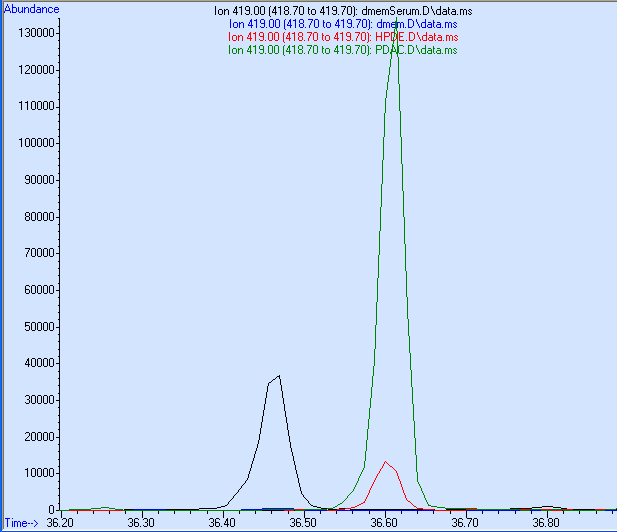  *absent in DMEM, present in serum  * produced and consumed |
| Aspartate 418  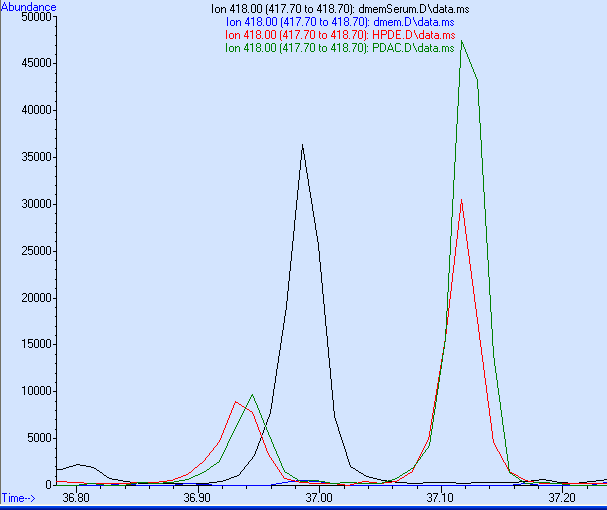  *absent in DMEM, present in serum | Glutamate 432  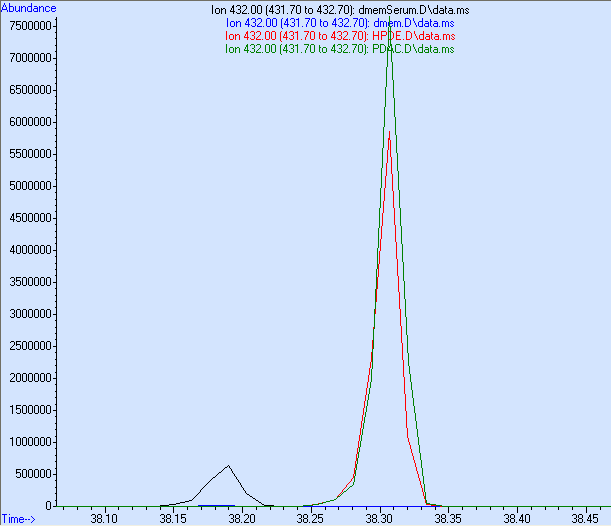  *absent in DMEM, present in serum  *produced |
| Glutamine 431  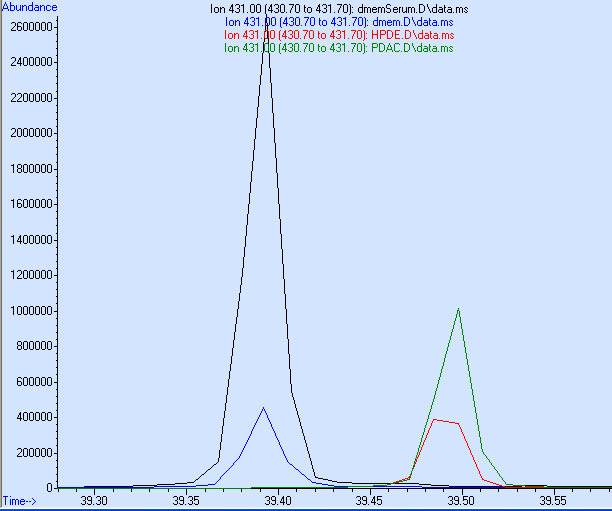  *present in DMEM, present in serum  *consumed | Citrate 591  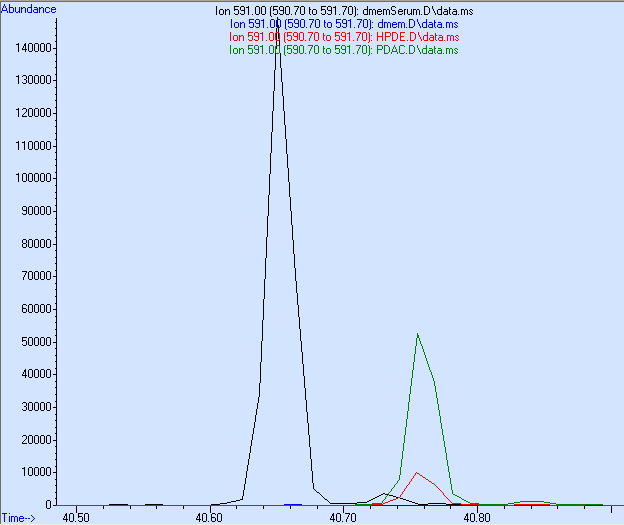  *present in serum  *consumed |

S3. GCMS analysis of low glucose DMEM derivatized by MOX-TMS

GCMS chromatogram of 20ul fresh DMEM containing 1 g/L glucose and 1mM sodium pyruvate obtained by SIM mode. Zoom-in windows show peaks of pyruvate (mz 174, black) and lactate (mz 219, blue) on the left panel, and glucose (mz 319, red) on the right panel. The 319 signal was saturated (>8,000,000). To analyse low glucose (1 g/L) DMEM required split injections due to disproportionate glucose and pyruvate signals. An alternative is to use 554 ion (m-15) for glucose, which consists of the full glucose backbone.

GCMS chromatogram of 20ul spent DMEM containing 1 g/L glucose and 1mM sodium pyruvate obtained by SCAN mode. Left panel shows full chromatogram; right panel shows chromatogram zoomed to pyruvate and lactate peaks. Ions 174, 175, 219 and 319 are shown. Right panel shows pyruvate peak (174) eluting closely to lactate peak (219), which contained high 175 ion.

A slow temperature programming (< 4 °C/min) was required to separate pyruvate and lactate peaks because they elute closely with each other shortly after the solvent front. Peak separation is critical because lactate, being the more abundant metabolite, interfered with the pyruvate 174 ion, contributing to a greater m+1 and m+2 signals.

S4. Methoximation-acetylation-butylation derivatization

GC chromatogram generated in SIM mode for 10 ul of fresh DMEM media spiked with 1 g/l [U-^13^C_6_]-glucose. Ions pairs 314:319 and 331:337 show relative enrichment of glucose at approximately 1:1 ratio for the C1-C5 and C1-C6 carbon backbones respectively.

One-pot derivatization of glucose, pyruvate and lactate using reagents methoxyamine, acetic anhydride and butanol. The methoximation step converts cyclic glucose into a linear form, and subsequent acetylation of glucose creates two products: methoxime glucose pentaacetate and gluconitrile pentaacetate. Carboxylic groups are activated using ethyl chloroformate before forming the corresponding butyl esters. All three synthesis steps are catalysed by pyridine. Dotted lines show possible fragmentation of analytes during electron impact ionization to produce ions of the corresponding masses (m/z). From the GC-MS spectra, we observed that the acetylation step also gave an unexpected by-product, gluconitrile-pentaacetate [[2](#_ENREF_2)]. The enrichment fractions of the full carbon backbone of glucose can be resolved using the glucose 331 ion [[1](#_ENREF_1)].

Pyruvate m1 and m2 signals omitted. Pyruvate (C7H12O2N1) signals from sample showed significantly higher m1 and m2 enrichment in samples. Chromatogram shows pyruvate ions 142 to 145. Left panel: standard; right panel: sample. Peak tailing may be reduced by exploring higher initial temperatures.


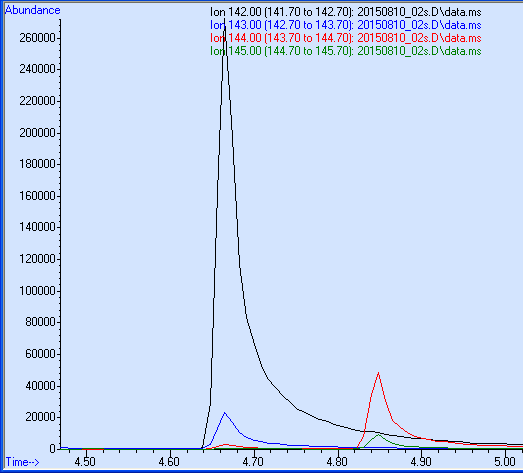

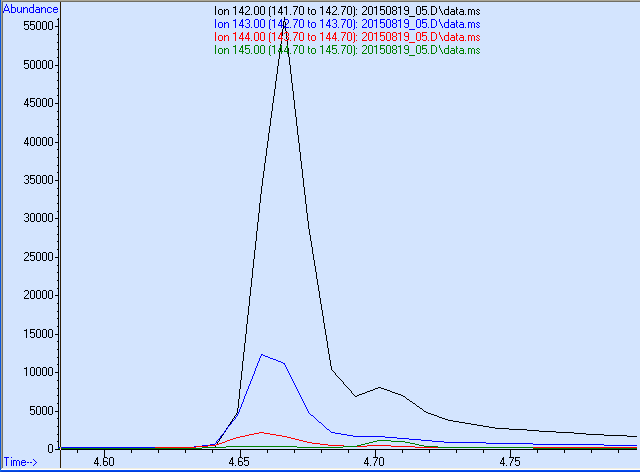


Mass isotopomer distribution of pyruvate

| mz | Standard | Sample | Theoretical |
| --- | --- | --- | --- |
| 142 (m0) | 0.9107 | 0.7706 | 0.9183 |
| 143 (m1) | 0.0751 | 0.1917 | 0.0749 |
| 144 (m2) | 0.0112 | 0.0319 | 0.0064 |
| 145 (m3) | 0.0031 | 0.0059 | 0.0004 |

S5. Flux analysis of FM and EM experiments

Flux results in the top panel were generated by performing Monte Carlo (50 interations) least-square analysis on FM and EM datasets separately. Glycolysis activity is represented by PK (pyruvate kinase) flux. Glutaminolysis activity is represented by ME1 (cytosolic malic enzyme) flux. The fraction of PK flux is shown at the bottom panel.

S6. Pathway carbon atom mapping

An Elementary Metabolite Unit (EMU) network was generated from the reaction network and the carbon atom transitions [[3](#_ENREF_3)]. Briefly, an EMU reaction shows the transfer of a group of reactant’s atoms to the product. Tracing is performed recursively from product to reactant, and ultimately atoms of a terminal metabolite of interest are mapped the corresponding atoms of the input substrates. Elementary mode analysis was then performed on the EMU reaction network to reveal pathway information contained in the 13C enrichment of pyruvate [[4](#_ENREF_4)]. Note that lactate contains the same information as pyruvate.

85404 unique modes to convert glucose (62612), glutamine (102) or both (22690) to pyruvate were generated from the metabolic model. This represents all possible conversion routes of glucose and/or glutamine carbon atoms to pyruvate. Unlike conventional reaction modes, these elementary EMU modes do not operate independently. For example, two pyruvate molecules are produced simultaneously from carbon 1-3 and 4-6 of glucose by EMP glycolysis, despite being described by two separate EMU modes.

The set of modes was interrogated by reaction participation—to identify the various atomic makeup of pyruvate carbon backbone based on reactions involved in its synthesis. Figure 6 illustrates all possible combinations of glucose or glutamine carbon atoms appearing in pyruvate given a set of active metabolic pathways. Notably, EMP glycolysis and partial glutaminolysis via cytoplasmic malic enzyme will solely produce pryuvate with intact 3-carbon backbone. PP pathway, without recycling, can produce pyruvate with intact 3-carbon backbone, but this comes as a mixture that includes intact 2-carbon backbone. A greater extent of PP pathway recycling produces a scrambling effect, resulting to various combinations of 2-carbon and individual carbon backbone. TCA cycle can also yield intact 2-carbon backbone, since acetyl-CoA backbone carbon is retained in the first cycle. One of the carbon pair is lost in the second cycle. While pyruvate is differentially labelled by EMP glycolysis, PP pathway, glutaminolysis and TCA cycle, these pathways produce overlapping pyruvate mass isotopomers. To resolve activities of these central carbon pathways by GCMS will therefore require different labelled substrates applied separately. For example, [1,2-13C2]-glucose for PP pathway, and [3,6-13C2]-glucose or [1,4-13C2]-glutamine for TCA cycle. To resolve the extent of recycling of fructose 6P and glyceraldehyde 3P through PP pathway, then [6-^13^C]-glucose should be used.

Here, we will use of a 1:1 uniformly labelled to unlabelled glucose mix and consider the expected outcomes of pyruvate enrichments under possible metabolic scenarios. Equally dominant m0 and m+3 pyruvate signals would indicate that glucose has been catabolised without the involvement of PP pathway or pyruvate dehydrogenase. The gain in m+1 and m+2 signals would indicate a greater PP pathway involvement in labelled glucose catabolism. These signals are further increased by recycling of PP pathway products, fructose-6-phosphate and glyceraldehyde-3-phosphate. Since pyruvate produced from PP pathway will have equal m+1 and m+2 signals due to reaction coupling, a greater m+1 signal relative to m+2 would suggest the involvement of the TCA cycle in producing pyruvate.

Following figures show mapping of glucose (G) and glutamine (Q) atoms to pyruvate given the engagement of a pathway to conversion the substrate to pyruvate. Left panel shows metabolic reactions engaged; middle panel shows conversion of substrate atoms to product; right panel shows the active EMU mappings in red edges.

1. Glycolysis

1. Glycolysis + TCA cycle with malic enzymes

1. Oxidative pentose-phosphate pathway without recycling

1. Oxidative pentose-phosphate pathway with recycling of fructose-6P and glyceraldehyde-3P

1. Glutaminolysis without pyruvate dehydrogenase

1. Glutaminolysis with pyruvate dehydrogenase

S7. Intracellular enrichment of PANC-1 cells at 10 hour

| pyruvate |  | control | tnfα |
| --- | --- | --- | --- |
|  | m0 | 0.560 | 0.581 |
|  | m1 | 0.031 | 0.025 |
|  | m2 | 0.016 | 0.013 |
|  | m3 | 0.393 | 0.380 |

| citrate |  | control | tnfα |
| --- | --- | --- | --- |
|  | m0 | 0.345 | 0.312 |
|  | m1 | 0.086 | 0.085 |
|  | m2 | 0.322 | 0.31 |
|  | m3 | 0.108 | 0.129 |
|  | m4 | 0.09 | 0.1 |
|  | m5 | 0.04 | 0.052 |
|  | m6 | 0.009 | 0.012 |

S8. OpenFLUX model for HEK 293 and PANC-1 cells

| RxnID | rxnEq | rxnCTrans | rates | rxnType | basis | deviation |
| --- | --- | --- | --- | --- | --- | --- |
| R01 | GLC_in = G6P | abcdef = abcdef |  | F | 100 |  |
| R02 | PYR = PYR_out | abc = abc |  | FR |  |  |
| R03 | PYR_in = PYR | abc = abc |  | R | X |  |
| R04 | LAC = LAC_out | abc = abc |  | FR | X |  |
| R05 | LAC_in = LAC | abc = abc |  | R | X |  |
| R06 | GLN_in = GLN | abcde = abcde |  | F |  |  |
| R07 | CO2 = CO2_out | a = a |  | F |  |  |
| R08 | G6P = F6P | abcdef = abcdef |  | FR |  |  |
| R09 | F6P = G6P | abcdef = abcdef |  | R | X |  |
| R10 | F6P = G3P + G3P | abcdef = cba + def |  | FR |  |  |
| R11 | G3P + G3P = F6P | cba + def = abcdef |  | R | X |  |
| R12 | G6P = R5P + CO2 | abcdef = bcdef + a |  | F | X |  |
| R13 | R5P + R5P = S7P + G3P | abcde + fghij = fgabcde + hij |  | FR |  |  |
| R14 | S7P + G3P = R5P + R5P | fgabcde + hij = abcde + fghij |  | R | X |  |
| R15 | S7P + G3P = E4P + F6P | abcdefg + hij = defg + abchij |  | FR |  |  |
| R16 | E4P + F6P = S7P + G3P | defg + abchij = abcdefg + hij |  | R | X |  |
| R17 | E4P + R5P = F6P + G3P | abcd + efghi = efabcd + ghi |  | FR |  |  |
| R18 | F6P + G3P = E4P + R5P | efabcd + ghi = abcd + efghi |  | R | X |  |
| R19 | G3P = PEP | abc = abc |  | F |  |  |
| R20 | PEP = PYR | abc = abc |  | F |  |  |
| R21 | PYR = LAC | abc = abc |  | FR |  |  |
| R22 | LAC = PYR | abc = abc |  | R | X |  |
| R23 | PYR = PYRm | abc = abc |  | F |  |  |
| R24 | PYRm = ACCOA + CO2 | abc = bc + a |  | F | X |  |
| R25 | ACCOA + OAA = CIT | ab + cdef = fedbac |  | F |  |  |
| R26 | CIT = AKG + CO2 | abcdef = abcde + f |  | F |  |  |
| R27 | AKG = 0.5 SUCC + 0.5 SUCC + CO2 | abcde = 0.5 bcde + 0.5 edcb + a |  | F |  |  |
| R28 | SUCC = MAL | abcd = abcd |  | F |  |  |
| R29 | MAL = OAA | abcd = abcd |  | F |  |  |
| R30 | MAL = PYRm + CO2 | abcd = abc + d |  | F | X |  |
| R31 | MAL = PYR + CO2 | abcd = abc + d |  | F | X |  |
| R32 | GLN = AKG | abcde = abcde |  | F |  |  |
|  |  |  |  |  |  |  |
| ## | excludedMetabolites |  |  |  |  |  |
| # | GLC_in |  |  |  |  |  |
| # | PYR_in |  |  |  |  |  |
| # | LAC_in |  |  |  |  |  |
| # | GLN_in |  |  |  |  |  |
| # | PYR_out |  |  |  |  |  |
| # | LAC_out |  |  |  |  |  |
| # | CO2_out |  |  |  |  |  |
| # | G6P_b |  |  |  |  |  |
|  |  |  |  |  |  |  |
| ## | simulatedMDVs |  |  |  |  |  |
| # | LAC_out#001 |  |  |  |  |  |
| # | LAC_out#011 |  |  |  |  |  |
| # | LAC_out#111 |  |  |  |  |  |
| # | PYR_out#001 |  |  |  |  |  |
| # | PYR_out#011 |  |  |  |  |  |
| # | PYR_out#111 |  |  |  |  |  |
|  |  |  |  |  |  |  |
| ## | inputSubstrates |  |  |  |  |  |
| # | GLC_in |  |  |  |  |  |
| # | PYR_in |  |  |  |  |  |
| # | LAC_in |  |  |  |  |  |
| # | GLN_in |  |  |  |  |  |

References

1. Schwender J, Ohlrogge JB, Shachar-Hill Y. A flux model of glycolysis and the oxidative pentosephosphate pathway in developing Brassica napus embryos. J Biol Chem. 2003;278:29442-53.

2. Price NP. Acylic sugar derivatives for GC/MS analysis of 13C-enrichment during carbohydrate metabolism. Anal Chem. 2004;76:6566-74.

3. Antoniewicz MR, Kelleher JK, Stephanopoulos G. Elementary metabolite units (EMU): a novel framework for modeling isotopic distributions. Metab Eng. 2007;9:68-86.

4. Pey J, Rubio A, Theodoropoulos C, Cascante M, Planes FJ. Integrating tracer-based metabolomics data and metabolic fluxes in a linear fashion via Elementary Carbon Modes. Metab Eng. 2012;14:344-53.
